# *Methylobacterium* spp. as Emerging Opportunistic Premise Plumbing Pathogens

**DOI:** 10.3390/pathogens9020149

**Published:** 2020-02-22

**Authors:** Kyle J. Szwetkowski, Joseph O. Falkinham

**Affiliations:** Department of Biological Sciences, Virginia Tech, Blacksburg, VA 24061, USA

**Keywords:** opportunistic pathogen, adherence, contact angle, biofilm, temperature susceptibility

## Abstract

*Methylobacterium* spp. are emerging opportunistic premise plumbing pathogens. Human infections linked to premise plumbing provide evidence of their routes of infection. Cells of a collection of representative strains of different *Methylobacterium* species were tested for hydrophobicity by contact angle, adherence and biofilm formation on different plumbing materials, and temperature tolerance (50–60 °C); characteristics shared by OPPPs. *Methylobacterium* spp. strains were shown to grow in drinking water, have high cell-surface hydrophobicity, adhere to pipe surface materials, form biofilms, and survive exposure to high (60° C) temperatures. It can be concluded that *Methylobacterium* spp. strains share traits in common with other opportunistic premise plumbing pathogens (OPPPs).

## 1. Introduction

Opportunistic premise plumbing pathogens (OPPPs) are normal microbial residents of drinking water, distribution systems, and premise plumbing [1] which are estimated to cause nearly 30,000 cases of human disease and cost $850 million a year [2]. OPPPs grow in drinking water distribution systems, unlike contaminants of drinking water such as *Escherichia coli* and *Salmonella* spp. Examples of OPPPs are: *Legionella pneumonia*, *Mycobacterium avium*, *Pseudomonas aeruginosa*, *Stenotrophomonas maltophilia*, and *Acinetobacter baumannii*. OPPPs share a number of traits impacting on their ecology, including growth at low oxygen (microaerobic) and organic matter content (oligotrophic), biofilm formation, and resistance to disinfectants [1]. Premise plumbing has a number of features that select for OPPPs, including low organic matter content, heating, periods of stagnation, and different pipe materials for biofilm formation. 

Members of the genus *Methylobacterium* are opportunistic pathogens of humans [3,4] found in water and soil. *Methylobacterium* spp. infections have been linked to patients with indwelling catheters [5] and pseudo-outbreaks associated with the use of bronchoscopes and endoscopes that were contaminated by tap water containing *Methylobacterium* spp. used for washing bronchoscopes [5,6]. Bloodstream infections have been reported due to *M. mesophilicum* from bone marrow transplant recipients caused by tap water used for oral irrigation [7]. In another case, a patient undergoing continuous ambulatory peritoneal dialysis developed a peritoneal infection due to *M. mesophilicum*, which originated from water in their bathroom [8]. In general, *Methylobacterium* spp. cause mild clinical symptoms such as fever, but are also responsible for bacteremia, peritonitis, and pneumonia [9,10]. *Methylobacterium* spp. produce pink carotenoid pigments as well as phytochromes (cytokinins and auxins), which are known to stimulate plant growth [11,12] and are capable of nitrogen fixation [13] and help plants to fight pathogens [14].

*Methylobacterium* spp. are found in a wide variety of natural habitats including soil, dust, air, fresh water, and aquatic sediments [15] and are normal inhabitants of drinking water distribution systems and premise plumbing. Specifically, high numbers of *Methylobacterium* spp. have been identified in shower curtains [16] and showerhead biofilms [17]. Interestingly, if *Mycobacterium* spp. were detected in showerhead biofilms, *Methylobacterium* spp. were absent, and vice-versa [17]. This observation was confirmed in a study of households containing patients with *Mycobacterium avium* pulmonary infections; when *Methylobacterium* spp. were present in biofilm samples, *M. avium* was absent [18]. The basis for that mutual exclusion appears to be based upon an inhibition of adherence of *M. avium* cells to biofilms of *Methylobacterium* spp. [19].

*Methylobacterium* spp. share a number of characteristics in common with OPPPs, including: chlorine resistance [20,21,22], biofilm formation [23,24], and desiccation tolerance [23,25]. *Methylobacterium* spp. are recognized as resistant to elevated (>50 °C) temperatures [26] and were isolated from hot tap water in a household [27], suggesting that the laboratory-measured temperature resistance [26] is of ecological consequence. Finally, in common with other OPPPs, *Methylobacterium extorquens* was isolated from amoebae in drinking water systems, making it an amoeba-resistant microorganism [28]. It is our objective to describe further characteristics of *Methylobacterium* spp. to reinforce the claim that they are opportunistic premise plumbing pathogens.

## 2. Results

### 2.1. Persistence of Methylobacterium spp. Strains in Tap Water

The survival of different *Methylobacterium* spp. strains in tap water was measured by inoculation of water-acclimated suspensions into sterile Blacksburg tap water. All strains survived the 21-day incubation period and all showed increased numbers after 7 days (Table 1). Notably, numbers of the *M. adhaesivum* strain doubled in 7 days and remained high throughout the 21-day incubation period (Table 1). It is likely that the fluctuation in survival values between time points reflect the aggregation and clumping of the strains (with the exception of *M. hispanicum* strain JM1-5.

### 2.2. Measurement of the Hydrophobicity of Methylobacterium spp. Cells on Different Plumbing Surfaces

Hydrophobicity, as reflected in the contact angle, provides a good estimate of bacterial cell surface hydrophobicity [29]. *Methylobacterium* spp. strains were grown in R2A broth at 30 °C with aeration (120 rpm) and acclimated to tap water. Lawns of a strain on each plumbing surface were prepared by incubating coupons at room temperature for six hours in the presence of 10^5^ CFU mL^−1^ of each strain in the CDC biofilm reactor. The coupons were removed, rinsed with sterile tap water, and contact angles were measured. The *Methylobacterium* spp. strains tested (triplicate replicates) produced higher contact angles than those of the control *Escherichia coli* on plumbing surfaces (Table 2). The highest contact angles were measured on PVC and galvanized coupons, but low contact angles were measured on glass (Table 2).

### 2.3. Measurement of Adherence of Methylobacterium spp. Cells to Plumbing Surfaces

Plumbing surface coupons (i.e., glass, copper, galvanized, PVC, and stainless steel) were exposed to water-acclimated *Methylobacterium* spp. cells at 10^5^ CFU mL^−1^ for 1–6 h in the CDC Biofilm Reactor to measure adherence. Cells of the *Methylobacterium* spp. strains rapidly adhered and reached high numbers, reaching between 350 and 3800 CFU cm^−2^ within 6 h (Table 3 and Table 4).

The values of adherent cells at 0 h reflect adherence occurring during the processing time for each coupon. The values for the adherent *Methylobacterium* spp. cells are likely underestimated, as 1%–10% of the cell numbers remained adhered to the coupons based on a detection of colonies after placing the vortexed coupons on R2A agar medium (independent experiment). There could be several reasons for the fact that the standard deviations of the triplicate measurements were higher for some samples (Table 3 and Table 4). If both single and aggregated cells adhered, there would be a wide range of adherent cells, or if aggregates adhered, the derived suspensions would consist of aggregates and single cells that would yield widely different colony counts depending on their ability to separate aggregates into single cells.

PVC and galvanized coupons had high numbers of adherent *M. extorquens* and *M. hispanicum* cells. The descending order of the *M. extorquens* strain’s adherence was: PVC, glass, galvanized, copper, and steel (Table 3), while that of the *M. hispanicum* strain’s adherence was: PVC, galvanized, steel, glass, and copper (Table 4). Based on these results, PVC and galvanized plumbing surfaces are likely to have the highest numbers of adherent *Methylobacterium* spp. cells.

### 2.4. Measurement of Biofilm Formation of Methylobacterium spp. on Plumbing Surfaces

Biofilm formation was measured as the increased number of adherent colony-forming units following adherence, washing the coupons free of non-adherent cells, and replacing them in the CDC biofilm device containing autoclaved Blacksburg tap water to minimize further adherence. The coupons were removed after 21 days and adherent CFU were counted. 

The results show, like adherence, plumbing material influenced biofilm formation. On both PVC and galvanized coupons, very high numbers of adherent *M. extorquens* cells were counted (Table 5). The descending order of biofilm numbers for *M. extorquens* was as follows: PVC, galvanized, copper, glass, and steel (Table 5).

### 2.5. Measurement of the Thermal Tolerance of Methylobacterium spp. Cells

To assess the thermal tolerance of *Methylobacterium* spp. at hot water-heater temperatures, survival measurements were performed after exposure to 50 °C, 55 °C, and 60 °C. Water-acclimated *Methylobacterium* spp. suspensions were diluted and exposed to each temperature for 1, 2, and 3 minutes. Survival of *Methylobacterium* spp. cells of all strains were relatively high after 3 min exposure to 50 °C (>80%) and 55 °C (20%–100%) (Data not shown). At 60 °C, *Methylobacterium* spp. strain survival ranged from 2% to 85%, substantially higher than the survival of *E. coli* (Table 6). All but the *Methylobacterium adhaesivum* strain were killed by 6 min exposure to 60 °C (Table 6). By extending the exposure at 60 °C to six minutes, the survival of the *M. adhaesivum* strain dropped to 8% (Table 6).

## 3. Discussion

The experimental approaches were devised to determine whether representative *Methylobacterium* spp. strains had characteristics found in OPPPs. The findings indicate *Methylobacterium* spp. are hydrophobic, survive in drinking water (some even growing in drinking water), adhere to many plumbing surfaces, form biofilms, and are relatively thermally tolerant. All these characteristics are shared by opportunistic premise plumbing pathogens [1].

New methods need to be developed to accurately measure the growth of the aggregating *Methylobacterium* spp. strains without disturbing their natural growth. *M. hispanicum* strain JM1-5 was the only strain that did not aggregate under laboratory conditions and thus provides the best measure of the variables reported here without the influence of aggregation.

Evidence that the *Methylobacterium* spp. strains survived and even grew in sterile tap water (Table 1) is in agreement with the discovery that *Methylobacterium* spp. were found to survive in autoclaved and filtered river water for up to 260 days [30]. The *Methylobacterium* spp. strain and the coupon type were major factors influencing surface adherence and biofilm formation. Hydrophobicity strongly influences surface colonization [31]. Therefore, it is not surprising that the number of adherent cells of the more hydrophobic and spontaneously aggregating *M. extorquens* strain was greater than those of the non-aggregating, less hydrophobic *M. hispanicum* strain JM1-5 (Table 3 and Table 4). Both aggregation and surface adherence are influenced by cellular hydrophobicity, as postulated by van Loosdrecht et al. [31]. The abilities of each *Methylobacterium* spp. strain to aggregate have been listed in Table 7. The materials in each plumbing surface also influenced the number of adherent cells. Galvanized (Zn) steel and PVC materials are generally hydrophobic surfaces, which leads to higher adherence and biofilm formation, while glass and steel are more hydrophilic surfaces, leading to less adherence and biofilm formation. There is speculation that high zinc concentrations in soil and water have also been associated with high mycobacterial numbers [32], and this could be related to the high number of adherent *Methylobacterium* spp. cells.

*Methylobacterium* spp. exhibited relative thermal tolerance at hot water temperatures (Table 6). Although killing was not seen until the temperatures were raised to 60 °C (Table 6), these results suggest that one way to reduce *Methylobacterium* spp. exposure is to elevate water heater temperatures to 60 °C. In a study of the numbers of nontuberculous mycobacteria (NTM) in household plumbing samples, it was found that NTM were less frequently recovered from household samples whose water heater temperatures were >130 °F (>55 °C) compared to ≤125 °F (≤50 °C) [33]. Other ways to reduce *Methylobacterium* spp. exposure in water heaters would be to drain and refill the water heater periodically.

In summary, *Methylobacterium* spp. share characteristics of other OPPPs, including persistence in tap water, hydrophobicity, an ability to aggregate, adhere, and form biofilms on different plumbing surfaces, and thermal tolerance [1]. Knowledge of the types of pipe material that support the lowest number of *Methylobacterium* spp. cells can be used to reduce *Methylobacterium* spp. colonization of plumbing in hospitals and homes. 

## 4. Materials and Methods 

### 4.1. Methylobacterium spp. Strains

The *Methylobacterium* spp. strains used in this study are listed in Table 7 and were chosen to be a range of species representative of the genus. 

### 4.2. Growth of Methylobacterium spp. Strains

For all experiments, strains were grown in 25 mL of R2A broth (High Media Laboratories, India) in a 250 mL Nephalometer flask to log phase with aeration (60 rpm) at 30 °C. As *Methylobacterium* spp. strains are likely to aggregate [4], cell number, turbidity (abs 540 nm), and cell protein [34] were measured. Comparison of measurements of absorbance at 540 nm and protein content of the non-aggregating strain *Methylobacterium hispanicum* strain JMI-5 demonstrated a strong correlation (R = 0.9841) [35].

### 4.3. Water Acclimation of Methylobacterium spp.

To produce *Methylobacterium* spp. cells whose characteristics would most likely resemble those of cells in drinking water, in distribution systems, and in premise plumbing, cells grown in RA2 broth were collected, washed twice, and suspended and incubated in sterile Blacksburg tap water for 7 days at room temperature [36]. Colony counts of the acclimated cells were measured on R2A. 

### 4.4. Biofilm Device

The CDC biofilm reactor (BioSurfaces Technologies Corporation, Bozeman, MT) was used with glass, steel, copper, PVC, and galvanized coupons [37]. Each coupon was 0.3 cm thick with a 1.27 cm diameter. The glass vessel allows for approximately 350 mL of operational fluid capacity and contains eight removable polypropylene rods, each capable of holding three coupons, which allows for 24 sampling opportunities. The use of the CDC biofilm reactor allows for an easy manipulation of biofilm growth surfaces, temperature, and shear [37].

### 4.5. Measurement of Hydrophobicity

Hydrophobicity, as reflected in the contact angle, provides a good estimate of bacterial cell surface hydrophobicity [29] and has a predictive value for adhesion [31]. Coupons in the CDC biofilm reactor were incubated at room temperature for six hours in the presence of 10^5^ CFU mL^−1^ of each strain for adherence. Rods with the coupons were removed from the paddle support system and rinsed by dipping three times in sterile Blacksburg tap water. Rods were allowed to air dry before recording measurements. Ten microliter drops of 1% saline were placed on cell biofilms on coupons in triplicate. The measurement of the contact angle was done by turning the rim of the goniometer until one of the cross-hairs was tangential to the drop at the place where it was in contact with the coupon’s surface [29].

### 4.6. Measurement of Adherence of Methylobacterium spp. Cells to Surfaces

Adherence of water-acclimated *Methylobacterium* spp. cells was measured in the CDC Biofilm Reactor as described [37].

### 4.7. Measurement of the Biofilm Formation of Adherent Methylobacterium spp. Cells

Biofilm formation by water-acclimated and adherent *Methylobacterium* spp. cells was measured in the CDC Biofilm Reactor as described [37].

### 4.8. Measurement of Temperature Susceptibility of Methylobacterium spp. Strains

The temperature of a water bath was set to 50 °C, 55 °C, or 60 °C. Water-adapted suspensions were diluted 1000-fold into Standard Hardness Water [38]. Two milliliters of the diluted suspension was transferred to a sterile 13 mm × 100 mm glass tube and 10 μL immediately spread on R2A agar in triplicate. At 1, 2, 3, and 6 minutes of exposure to each temperature, 10 μL of suspension was spread on R2A agar in triplicate. Plates were incubated at 30 °C for five days and colonies were counted to calculate survival as a percentage of the non-heat-exposed control.

## Figures and Tables

**Table 1:** Persistence of *Methylobacterium* spp. strains in tap water.

Strain	Percent Survival after *		
	7 Days	14 Days	21 Days
*M. extorquens*	130	58	63
*M. aquaticum*	236	100	73
*M. adhaesivum*	200	225	250
*M. isbiliense*	140	90	100
*M. variable*	181	55	73
*M. hispanicum* JM 1-5	114	90	94
*M. hispanicum* JM1-8	200	75	83

* An average of cells at 7, 14, or 21 days divided by average at inoculation expressed as a percentage.

**Table 2:** Contact angles (degrees) of *Methylobacterium* spp. and *Escherichia coli* cells on different plumbing surfaces *.

Strain	Steel	PVC	Galvanized	Copper	Glass
No Cells	58.0 ± 5.3	97.0 ± 5.9	85.4 ± 5.0	66.5 ± 9.8	35.3 ± 2.7
*M. hispanicum* JM1-5	63.9 ± 3.7	83.7 ± 3.1	86.5 ± 1.9	70.4 ± 2.6	48.2 ± 2.7
*E. coli*	58.6 ± 3.3	66.1 ± 1.5	74.5 ± 3.6	49.4 ± 11.0	47.8 ± 3.5
*M. extorquens*	65.6 ± 3.1	97.3 ± 5.3	93.3 ± 4.6	71.0 ± 2.7	50.8 ± 4.6

* Average contact angle ± standard deviation of adhering cells to each coupon type of triplicate measurements.

**Table 3:** Adherence of *Methylobacterium* extorquens strain ATCC 43645 cells to plumbing surfaces *.

Duration (h)	CFU cm^−2^ Surface				
	Steel	Copper	PVC	Galvanized	Glass
0	180 ± 10	250 ± 3	1100 ± 90	640 ± 40	270 ± 4
1	460 ± 20	650 ± 30	1900 ± 70	1300 ± 30	1500 ± 90
2	680 ± 20	1000 ± 20	2200 ± 40	870 ± 30	1900 ± 30
3	870 ± 50	1300 ± 10	2100 ± 30	2000 ± 50	1900 ± 30
6	1100 ± 50	2000 ± 20	3800 ± 30	3000 ± 50	3800 ± 20

* Average number of CFU/cm^2^ ± standard deviation adhering to each coupon type of triplicate measurements.

**Table 4:** Adherence of *Methylobacterium hispanicum* strain JM1-5 to plumbing surfaces *.

Duration (h)	CFU cm^−2^ on				
	Steel	Copper	PVC	Galvanized	Glass
0	160 ± 6	80 ± 8	110 ± 20	200 ± 10	8 ± 1
1	560 ± 50	40 ± 4	1000 ± 80	1400 ± 20	120 ± 4
2	480 ± 30	100 ± 2	430 ± 20	2400 ± 30	210 ± 4
3	390 ± 6	200 ± 10	580 ± 7	1100 ± 70	220 ± 10
6	870 ± 30	350 ± 30	1600 ± 110	1400 ± 50	450 ± 20

* Average number of CFU/cm^2^ ± standard deviation adhering to each coupon type of triplicate measurements.

**Table 5:** Biofilm formation on plumbing surfaces by *M. extorquens*.

Duration	CFU cm^−2^ Surface *				
	Steel	Copper	PVC	Galvanized	Glass
6 h (adherence)	110 ± 10	250 ± 20	1100 ± 340	670 ± 120	270 ± 56
21 days	310 ± 67	9200 ± 230	620,000 ± 970	450,000 ± 980	2200 ± 540

* Average number of adherence CFU cm^−2^ ± standard deviation from triplicate measurements.

**Table 6:** Survival of *Methylobacterium* spp. strains at 60 °C.

Strain	Percent Survival at 60 °C	
	3 min	6 min
*M. extorquens*	2	<1
*M. aquaticum*	18	<1
*M. adhaesivum*	85	8
*M. isbiliense*	18	<1
*M. variable*	2	<1
*M. hispanicum* JM1-5	5	<1
*M. hispanicum* JM1-8	6	2
*E. coli*	<1	<1

**Table 7:** *Methylobacterium* spp. strains used in this study.

Species	Strain	Source	Aggregation
*M. extorquens*	ATCC 43645	Soil, Japan	Yes
*M. aquaticum*	NCIMB 14006	Drinking Water, Spain	Yes
*M. adhaesivum*	NCIMB 14625	Drinking Water, Spain	Yes
*M. isbiliense*	NCIMB 14626	Drinking Water, Spain	Yes
*M. variable*	NCIMB14628	Drinking Water, Spain	Yes
*M. hispanicum*	JM1-5	Shower Water, Virginia	No
*M. hispanicum*	JM1-8	Shower Water, Virginia	Yes

## References

[1] Falkinham J.O. (2015). Common features of opportunistic premise plumbing pathogens. Int. J. Environ. Res. Public Health.

[2] Collier S.A., Stockman L.J., Hicks L.A., Garrison L.E., Zhou F.J., Beach M.J. (2012). Direct healthcare costs of selected diseases primarily or partially transmitted by water. Epidemiol. Infect..

[3] Kressel A.B., Kidd F. (2001). Pseudo-outbreaks of *Mycobacterium chelonae* and *Methylobacterium mesophilicum* caused by contamination of an automated endoscopy washer. Infect. Control Hosp. Epidemiol..

[4] Gallegos V., Garcia M.T., Ventosa A. (2006). *Methylobacterium adhaesivum* sp. nov., a methylotrophic bacterium isolated from drinking water. Int. J. Syst. Evol. Microbiol..

[5] Poirier A., Lapointe R., Claveau S., Joly J.R. (1988). Bacteremia caused by *Pseudomonas mesophilica*.. Can. Med. Assoc. J..

[6] Flourney D.J., Petrone R.L., Voth D.W. (1992). A pseudo-outbreak of *Methylobacterium mesophilica* isolated from patients undergoing bronchoscopy. Eur. J. Clin. Microbiol. Infect. Dis..

[7] Gilchrist M.J., Kraft J.A., Hammond J.G., Connelly B.L., Myers M.G. (1986). Detection of *Pseudomonas mesophilica* as a source of nosocomial infections in a bone marrow transplant unit. J. Clin. Microbiol..

[8] Rutherford P.C., Narkowicz J.E., Wood C.J., Peel M.M. (1988). Peritonitis caused by *Pseudomonas mesophilica* in a patient undergoing continuous ambulatory peritoneal dialysis. J. Clin. Microbiol..

[9] Sanders J.W., Martin J.W., Hooke M., Hooke J. (2000). *Methylobacterium mesophilicum* infection: Case report and literature review of an unusual opportunistic pathogen. Clin. Infect. Dis..

[10] Lai C.C., Cheng A., Liu W.L., Tan C.K., Huang Y.T., Chung K.P., Lee M.R., Hsueh P.R. (2011). Infections caused by unusual *Methylobacterium* species. J. Clin. Microbiol..

[11] Ivanova E.G., Doronina N.V., Trotsenko Y.A. (2001). Aerobic methylobacteria are capable of synthesizing auxins. Microbiologiya.

[12] Koeing R.L., Morris R.O., Polacco J.C. (2002). tRNA is the source of low-level *trans*-zeatin production in *Methylobacterium* spp.. J. Bacteriol..

[13] Sy A., Giraud E., Jourand P. (2001). Methylotrophic *Methylobacterium* bacteria nodulate and fix nitrogen in symbiosis with legumes. J. Bacteriol..

[14] Holland M.A., Polacco J.C. (1994). PPFMS and other covert contaminants: Is there more to plant physiology than just plant?. Annu. Rev. Plant Physiol. Plant Mol. Biol..

[15] Hirashi A., Furuhata K., Matsumoto A., Koike K.A., Fukuyama M., Tabuchi K. (1995). Phenotypic and genetic diversity of chlorine-resistant *Methylobacterium* strains isolated from various environments. Appl. Environ. Microbiol..

[16] Kelley S.T., Theisen U., Angenent L.T., Amand A.S., Pace N.R. (2004). Molecular analysis of shower curtain biofilm microbes. Appl. Environ. Microbiol..

[17] Feazel L.M., Baumgartner L.K., Peterson K.L., Frank D.N., Harris J.K., Pace N.R. (2009). Opportunistic pathogens enriched in showerhead biofilms. Proc. Natl. Acad. Sci. USA.

[18] Falkinham J.O.I.I.I., Williams M.D., Kwait R., Lande L. (2016). *Methylobacterium* spp. as an indicator for the presence or absence of *Mycobacterium* spp.. Int. J. Mycobacteriol..

[19] Munoz-Egea M.C., Ji P., Pruden A., Falkinham J.O. (2015). Exclusion of *Mycobacterium avium* from household plumbing biofilms by *Methylobacterium* spp.. Pathogens.

[20] Furuhata K., Koike K.A., Matsumoto A. (1989). Growth and survival of a chlorine resistive gram-negative rod bacterium, *Protomonas extorquens* isolated dominantly from drinking tank-water. Bull. Jpn. Soc. Microbiol. Ecol..

[21] Furuhata K., Koike K.A. (1993). Isolation of *Methylobacterium* spp. from drinking tank-water and resistance of isolates to chlorine. Koshuu.

[22] Furuhata K., Banzai A.U., Kawakami Y., Ishizaki N., Yoshida Y., Goto K., Fukuyama M. (2011). Genotyping and chlorine-resistance of *Methylobacterium aquaticum* isolated from water samples in Japan. Biocontrol Sci..

[23] Yano T., Kubota H., Hanai J., Hitomi J., Tokuda H. (2013). Stress Tolerance of *Methylobacterium* biofilms in bathrooms. Microbes. Environ..

[24] Simoes L.C., Simoes M., Vieira M.J. (2009). Susceptibility of drinking water biofilm bacteria to sodium hypochlorite. http://repositorium.sdum.uminho.pt/bitstream/1822/9607/1/EUROBIOFILMS2009_Abstract-Simoes%5B1%5D.pdf.

[25] Kaneko M., Hiraishi A. (1991). Physico-chemical and microbiological research on pink-colored aggregates found in a potable water treatment and distribution system. J. Jpn. Water Works Assoc..

[26] Orphan V.J., Taylor L.T., Hafenbradl D., Delong E.F. (1999). Culture-dependent and culture-independent characterization of microbial assemblages associated with high-temperature petroleum reservoirs. Appl. Environ. Microbiol..

[27] Soucie W.J., Schuler B. (2006). Avoiding pink stain pain. Am. Water Works Assoc..

[28] Thomas V., Herrera-Rimann K., Blanc D.S., Greub G. (2006). Biodiversity of amoebae and amoebae-resisting bacteria in a hospital water network. Appl. Environ. Microbiol..

[29] van Oss C.J., Gillman C.F., Neumann A.W. (1975). Phagocytic Engulfment and Cell Adhesiveness as Cellular Phenomena.

[30] Flint K.P. (1987). The long term-survival of *Escherichia coli* in river water. J. Appl. Bacteriol..

[31] Van Loosdrecht M.C., Lyklema J., Norde W., Schraa G., Zehnder A.J. (1987). The role of bacterial cell wall hydrophobicity in adhesion. Appl. Environ. Microbiol..

[32] Kirschner R.A., Parker B.C., Falkinham J.O. (1992). Epidemiology of infection of nontuberculous mycobacteria. X. *Mycobacterium avium, M. intracellulare,* and *M. scrofulaceum* in acid, brown swamps of the southeastern United States and their association with environmental variables. Am. Rev. Respir. Dis..

[33] Falkinham J.O. (2011). Nontuberculous mycobacteria from household plumbing of patients with nontuberculous mycobacteria disease. Emerg. Infect. Dis..

[34] Lowry O.H., Rosebrough N.J., Farr A.L., Randall R.J. (1951). Protein measurement with the Folin-phenol reagent. J. Biol. Chem..

[35] Falkinham J.O. (2003). Factors influencing the chlorine susceptibility of Mycobacterium avium, Mycobacterium intracellulare, and Mycobacterium scrofulaceum. Appl. Environ. Microbiol..

[36] Goeres D.M., Loetterle L.R., Hamilton M.A., Murga R., Kirby D.W., Donlan R.M. (2005). Statistical assessment of a laboratory method for growing biofilms. Microbiology.

[37] Mullis S.N., Falkinham J.O. (2013). Adherence and biofilm formation of *Mycobacterium avium, Mycobacterium intracellulare*, and *Mycobacterium abscessus* to household plumbing materials. J. Appl. Microbiol..

[38] Schulze-Röbbecke R., Buchholtz K. (1992). Heat susceptibility of aquatic mycobacteria. Appl. Environ. Microbiol..

